# Influence of oviposition-inducing hormone on spawning and mortality in the endangered Panamanian golden frog (*Atelopus zeteki*)

**DOI:** 10.1186/s40850-021-00076-8

**Published:** 2021-05-22

**Authors:** Ellen Bronson, Emmet L. Guy, Kevin J. Murphy, Kevin Barrett, Andrew J. Kouba, Vicky Poole, Carrie K. Kouba

**Affiliations:** 1 Maryland Zoo in Baltimore, 1876 Mansion House Drive, Baltimore, MD 21217 USA; 2 Department of Biochemistry, Molecular Biology, Entomology, and Plant Pathology, Mississippi State University, Mississippi State, MS 39762 USA; 3 Department of Wildlife, Fisheries, and Aquaculture, Mississippi State University, Mississippi State, MS 39762 USA; 4 Department of Ectotherms, Fort Worth Zoo, Fort Worth, TX 76110 USA

**Keywords:** Anuran reproduction, Dystocia, Dopamine agonist, GnRHa, Metoclopramide, Hormone pulsing, Priming, Spawning

## Abstract

**Background:**

With Panamanian golden frogs (*Atelopus zeteki*; PGFs) likely extirpated from the wild, ensuring long-term sustainability of captive populations is crucial in order to conserve this critically endangered species. Unfortunately, PGFs display a unique reproductive behavior involving a prolonged period of amplexus leading to challenges in their successful captive propagation. The Maryland Zoo in Baltimore has observed high levels of mortality during the breeding season and suboptimal reproductive success leading to the use of hormone stimulation to aid in reproduction and health management.

**Methods:**

This project aimed to develop induced ovulation and health management protocols by (1) evaluating different doses of gonadotropin releasing hormone analogue (GnRHa), (2) comparing the efficacy of GnRHa and GnRHa + metoclopramide, (3) determining latency periods and the effects of pulsed hormone sequences; and (4) establish if mortality is impacted by hormone therapy. Female PGFs (*n* = 174) were given GnRHa either in various concentrations (Experiment 1) or combined with metoclopramide (Experiment 2), and oviposition success, latency, and mortality were measured as binary response variables.

**Results:**

Overall, the use of exogenous hormones significantly decreased mortality when compared to the control data of natural egg-laying females. GnRHa doses of 0.05 μg/g body weight produced similar ovulation rates compared to higher doses, and the addition of metoclopramide did not increase oviposition success compared to GnRHa alone. Lastly, results indicate the majority of female PGFs will release eggs within 48 h following the initial pulse of hormones with a small percentage ovipositing after a second pulse.

**Conclusion:**

Findings from this study will benefit captive management of PGFs by documenting the increased survival of females when given hormone stimulation and defining appropriate GnRHa doses and expected latency to spawning.

## Background

The Panamanian golden frog (PGF; *Atelopus zeteki*) is a critically endangered amphibian [1] whose historical wild populations were decimated by the global amphibian disease *Batrachochytrium dendrobatidis* (*Bd*) [2–6]. In 2006, as a response to their rapidly declining numbers, captive assurance colonies were established in Panama and several North American zoos and aquaria, with Maryland Zoo in Baltimore (MZIB) holding the largest number of animals [7, 8]. The PGF has not been seen in the wild since 2009 (E. Griffith, *pers comm*.), and with the PGF population presumed to be exclusively in captivity, maximal reproductive output with optimal genetic sustainability and minimal risk to existing individuals is crucial for species survival. Management efforts to promote captive breeding capitalize on knowledge of natural breeding behavior and reproductive cycles [9, 10]; however, little data was able to be collected on this species before it disappeared from its native range [9, 11]. Several *Atelopus* species display a unique reproductive behavior, in that amplexus is initiated reliably by the male and lasts for extended periods of time, sometimes up to 60 days or more [12], which is thought to stem from the necessity of males to competitively monopolize females at natural breeding sites until they oviposit [11, 13]. In captivity, extended periods of amplexus result in lack of food intake for several weeks, which depletes existing energy stores, causes poor body condition and can compromise the frog and make it more susceptible to systemic infection, all which contribute to increased risk of mortality [14–16]. These behavioral and physiologic responses are similar to observations in the widely recognized reproductive disorder ‘egg dystocia’ described for reptiles [17], and threaten the success of the PGF assurance colonies.

The high level of morbidity and mortality in the captive population of PGFs primarily occurs during the breeding season, which appears to be related to the failure of gravid females to undergo oviposition [15, 16]. Between 2009 and 2014, 67% of adult frogs and 72% of adult females over 2 yrs. of age that died anually in the Maryland Zoo’s PGF colony, expired during the breeding season (November–May) [16]. For other ectothermic species such as reptiles, health concerns related to dystocia can often be circumvented through hormone therapy, such as the use of oxytocin to stimulate oviduct contractions in turtles [18]. Similarly, exogenous hormones have been used to promote oocyte development and spawning in amphibians [19–27]. The development of an effective exogenous hormone therapy to stimulate spawning for the PGF population would have several potential benefits including increased reproductive output, less time in amplexus, reduced mortality, and the acquisition of more knowledge about this species’ unique reproductive biology in order to optimize care.

Natural oviposition results from environmental stimuli triggering the neuroendocrine cascade of the hypothalamus-pituitary-gonad axis (HPG-axis), whereby endogenous gonadotropin releasing hormone (GnRH) causes release of luteinizing hormone (LH) and follicle-stimulating hormone (FSH) [14, 28, 29]. Steroid sex hormones produced by the ovary lead to follicle maturation, vitellogenesis, oocyte maturation and spawning [30–32]. However, the natural endogenous cascade of hormones that impact these reproductive processes are often impaired in captive animals due to missing environmental cues. To circumvent the need for external breeding signals, exogenous hormones, such as gonadotropin releasing hormone analog (GnRHa), can be administered as a means of hormone therapy. The exogenous GnRHa acts on the HPG-axis at the anterior pituitary activating the natural hormone cascade and production of LH/FSH to stimulate steroidogenesis in the gonads [21, 30, 31, 33–35].

Determining the optimal hormone regimen is a critical step for promoting gametogenesis and spawning, but responses to specific hormones, their combinations, or doses vary widely across species [14, 34]. For example, GnRHa induces ovulation when administered at 0.4 μg/g body weight (BW) for many amphibian species, but responses to a wide range of GnRHa concentrations (0.1–2 μg/g BW) have also been reported [20, 23, 27, 34, 36, 37]. Amphiplex, which is a combined mixture of GnRHa plus the dopamine antagonist metoclopramide (MET), has been used to induce spawning in Northern leopard frogs (*Lithobates pipiens*) [21, 38] and dusky gopher frogs (*Lithobates sevosa)* [24], as well as spermatogenesis in male PGFs [39]. The addition of MET is proposed to limit dopamine inhibition of GnRH, thus enhancing LH/FSH release and gametogenesis [21, 38]. Although Amphiplex has been shown to benefit male PGF sperm production, it is unclear whether the combined hormone mixture would increase female spawning rates above GnRHa administered alone.

Depending on the species and the stage of gametogenesis, a single exogenous hormone stimulation can often successfully induce ovulation. However, there are amphibian species in which priming or pulses of hormones are required to facilitate the spawning process. The beneficial impacts of primed or pulsed hormones on increased spawning rates have been described for Wyoming toad (*Anaxyrus baxteri)* [19], Fowler’s toad (*Anaxyrus fowleri*) [25], Günther’s toadlet (*Pseudophryne guentheri*) [22], corroboree frog (*Pseudophryne corroboree*) [20], dusky gopher frog [24, 40], Puerto Rican crested toad (*Peltophryne lemur*) [37] and Chiricahua leopard frog *(Lithobates chiricahuensis)* (Kouba per. communication). Previous studies by our lab on hormone priming effects on spawning rates [19, 24, 25, 40] were primarily designed to bypass unknown environmental cues necessary for ovulation, which are often required to complete oogenesis and vitellogenesis, once oocytes are arrested at the diplotene stage of meiotic prophase. Thus, hormone priming is meant to complete oocyte maturation, while pulsed hormones increase spawning rates by capitalizing on a two-phased process similar to that seen in nature. The first pulse causes all mature eggs to ovulate; whereas, for immature primary oocytes, a resumption of meiosis occurs due to progesterone synthesis by the follicle cells in response to the gonadotropic hormones produced by the pituitary gland following exogenous GnRHa administration [41]. A second pulse of hormone 24–48 h later initiates the first meiotic division of chromosomes from the pool of recruited primary oocytes and ovulation of another round of mature ovum from the ovary occurs. Examining pulsatile effects on female PGF spawning rates would provide critical insight into the timing of this species’ reproductive processes, which are already deemed unusual as reflected by the extended amplexus period.

The goal of this study was to develop an effective hormone protocol for induction of ovulation and spawning in captive female PGFs that serves to both enhance assisted breeding as well as alleviate the risk of death from egg binding. To address both these issues, the objectives of this study were to: (1) evaluate different doses of GnRHa, (2) compare the efficacy of GnRHa and GnRHa + MET, (3) determine latency periods and the effects of pulsed hormone sequences; and (4) establish if mortality is impacted by hormone therapy. Since the MZIB’s breeding program uses hormone stimulation as a method for maintaining both the health and reproduction of female frogs, this study focused primarily on number of spawning females and survival as the end goal and does not attempt to quantify the effects different hormone regimens have on clutch size, fertilizing capacity, or larval development.

## Results

### Natural spawning background rates

The control data set was obtained from historic PGF breeding records collected between 2009 and 2016 on all females (*n =* 431) allowed to breed naturally. Naturally breeding females were those that spawned without hormones in the first 2 weeks of the natural breeding period or which never received hormones and were allowed to continue natural breeding past the 2-weeks for up to 60 days. These extended natural breeding instances occurred primarily in the early breeding seasons 2009–2011. In total, 102 females laid eggs without hormone therapy in this 7-year time frame. Within the group of females that were amplexed by a male and naturally spawned, 23% laid eggs within 24 h, 45% within 1–7 days, 22% within 8–14 days and 10% past 2 weeks. For the majority of natural spawning incidences, 80% of those cases occurred during the early breeding years between 2009 and 2011. Only eight females laid eggs naturally within the first 2 weeks of amplexus for the two seasons spanning 2014–2016, when our two experiments were conducted. We observed a decreasing trend in reproductive capacity over time from 2009 to 2016, possibly due to extended time in captivity or hatching of new animals into captive settings with no residual knowledge of natural stimuli. Females that did not naturally spawn within the first 2 weeks of amplexus, during the 2014–2016 reproductive season, were used for hormone-induced ovulation Experiments 1 and 2 described below.

Mortality rates were determined for naturally breeding females over the same 7-year time frame to establish the background death rate in spawning and non-spawning females. The background mortality rate of females that successfully spawned was 11.7% but the mortality rate of non-spawning females that were never treated with hormones and died within 60 days of amplexus was 89%. Steep mortality rates of non-spawning females in years prior to 2009 were motivation for application of exogenous hormone therapy techniques, and following the 2009–2010 season, nearly all females were treated with a default regimen of 0.4 μg GnRHa after 2 weeks of natural breeding attempts failed. Deaths reported here in non-spawning females came from females in early breeding years and those that died during the 2-week natural breeding window.

### Experiment 1: GnRHa concentration response

The efficacy of GnRHa at four concentrations was tested to determine the optimum dosage needed to achieve efficient spawning in the PGF. Females laid eggs at a response rate of 65.2% (*n =* 15/23), 48.0% (*n =* 12/25), 59.1% (*n =* 13/22), and 45.0% (*n =* 9/20) when given GnRHa concentrations of 0.05, 0.1, 0.2, and 0.4, μg/g BW, respectively (Table 1). A chi-squared test of independence showed no significant effect of concentration, (χ^2^ (3, 90) = 2.406, *p* = .49). Although nearly 20% more females spawned when given 0.05 compared to 0.4 μg/g BW, the effect was not significant (χ^2^ (1, 43) = 1.773, *p* = .18). Overall, hormone therapy using GnRHa at any concentration between 0.05–0.4 μg/g BW can induce spawning above the rate of untreated controls, and with equal efficacy.

**Table 1:** Oviposition by female Panamanian golden frogs (*Atelopus zeteki*) given GnRHa treatments of 0.05, 0.1, 0.2, or 0.4 μg/g body weight. Data reflects the number and percent (%) of treated females that laid eggs, and those that did not lay eggs

**Treatment**	**N**	# **Females Ovipositing (%)**			**Did not lay**
No hormone	431	102 (23.7)			329 (76.3)
All GnRHa doses	90	49 (54.4)			41 (45.6)
*P*-value	–	0.00002			–
**GnRHa μg/g BW**	N	# **Females Ovipositing (%)**	**Pulse Response (%)**		**Did not lay**
			Laid w/ 1 pulse	Laid w/ 2 pulses	
0.05	23	15 (65.2)	11 (47.8)	4 (17.4)	8 (34.8)
0.10	25	12 (48.0)	10 (40.0)	2 (8.0)	13 (52.0)
0.20	22	13 (59.1)	11 (50.0)	2 (9.1)	9 (40.9)
0.40	20	9 (45.0)	8 (40.0)	1 (5.0)	11 (55.0)
P-value	–	0.49	0.86	0.41	0.49

There was no significant difference between the various GnRHa treatments on number of spawning females, which allowed us to combine all hormone treatment animals together (*n* = 90) and compare to the control (*n* = 431), where animals were allowed to naturally spawn for 2 weeks. Overall, treatment of female PGFs with GnRHa elicited a significantly higher (χ^2^ (1, 521) = 34.27, *p* = .00002) spawning response compared to natural spawning controls (Table 1). Only 23.7% (102/431) of female PGFs spawned when allowed to breed without hormone therapy, while 54.4% (49/90) of those treated with GnRHa laid eggs. The spawning response to GnRHa is even more pronounced within the experimental period of 2014–2016, where only 4.4% (8/182) of females spawned in the 2-week natural breeding window (data not shown). All PGF females in Experiment 1 exhibited either a gravidity grade of 5 (77.8%, *n =* 70/90) or 4 (22.2%; *n =* 20), upon being paired with a male. No effect of gravidity (χ^2^ (1, 90) = 1.029, *p* = .31) between grades 4 or 5 was found for ovipositing females in response to hormone treatment.

The overall spawning rate includes females receiving either one or two pulses of hormone at the given concentration of GnRHa (Table 1). The spawning response after one hormone pulse was similar for all concentrations tested, (χ^2^ (3, 90) = 0.742, *p* = .86), such that 47.8% (*n =* 11/23), 40.0% (*n =* 10/25), 50.0% (*n* = 11/22), and 40.0% (*n =* 8/20) of all females laid eggs when given GnRHa dosages of 0.05, 0.1, 0.2, and 0.4 μg/g BW, respectively. The second pulse of GnRHa (at the same concentration) induced a smaller proportion of the remaining females (*n =* 50) to oviposit, specifically 33.3% (*n =* 4/12), 13.0% (*n =* 2/15), 18.2% (*n =* 2/11), and 8.3% (*n =* 1/12), and also with no effect of concentration (χ^2^ (3, 50) = 2.893, *p* = .41). In addition, we examined the response based on the cumulative amount of hormone received from all pulses and also found no difference (χ^2^ (4, 139) = 5.544, *p* = .24), although a general inverse-trend was observed. For instance, the proportion of females ovipositing decreased (47.8, 37.8, 35.1, 29.0 and 9.1%) as the *cumulative* amount of GnRHa (sum of GnRHa treatments, each ranging from 0.05–0.4 μg/g BW, in 1 or 2 pulses depending on response rate), required to induce oviposition, increased (0.05, 0.1, 0.2, 0.4 and 0.8 μg/g BW), data not shown. Thus, spawning can be induced using the lowest tested GnRHa concentration of 0.05 μg/g BW (1 or 2 pulses); whereas, higher concentrations of GnRHa, either as 1 or 2 pulses, did not increase the overall percent of the population that will lay eggs.

**Fig. 1:**
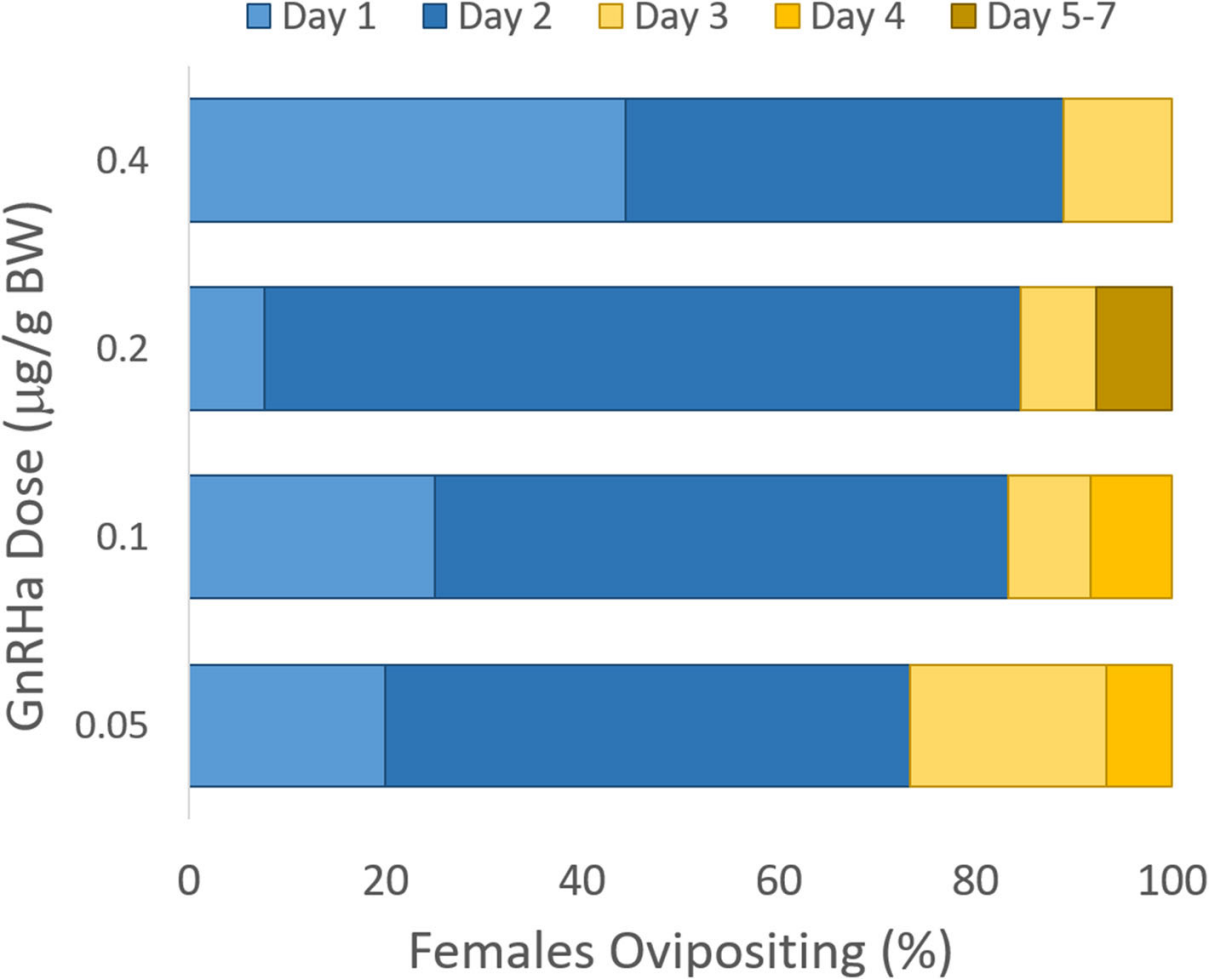
Within the group of ovipositing female PGFs, the latency in days to oviposition after one (blue shades) or two (gold shades) pulses of GnRHa, at 4 different concentrations

Even though no difference was observed in the percent of females responding to increasing concentrations of GnRHa, there was an inverse trend in the latency to oviposition within the sub-population of females that did lay eggs (Fig. 1). Of the successfully spawning female PGFs given 0.4 μg/g, 88.8% (*n =* 8/9) dropped eggs after the first pulse, compared to 73.3% (*n =* 11/15) when given 0.05 μg/g (Table 1, Fig. 1). Generally, as the dosage of GnRHa increased, more of the females laid eggs at earlier time points after initiation of hormone therapy. Within each treatment group, the highest proportion of females 53.3, 58.3, 76.9 and 44.4%, spawned between 24 and 48 h of the initial pulse for concentrations of 0.05, 0.1, 0.2, and 0.4 μg/g BW, respectively (Fig. 1). After a 2nd hormone pulse, some females receiving 0.05–0.2 μg/g BW required > 72 h to lay eggs, whereas at a concentration of 0.4 μg/g BW all oviposition was complete by 72 h (Fig. 1). Combined, these observations indicate that higher doses of GnRHa may induce females to lay eggs sooner compared to lower doses, albeit the effect was not significant in this trial.

Finally, we compared the rate of mortality across GnRHa concentrations and the number of hormone pulses administered to determine if there was an unforeseen risk in using hormone concentrations in this range compared to control. The mortality rate for hormone treated females was no different than the background mortality of non-treated females (χ^2^ (1, 201) = 0.551, *p* = .46) (Table 2), indicating that hormone treatment did not subject female PGFs to an additional risk of mortality beyond the natural background rate. Spawning females were similarly at risk of death whether they underwent hormone therapy (16.3%; *n =* 8/49) or laid eggs naturally (11.7%; *n =* 12/102), (χ^2^ (1, 151) = 0.599, *p* = .44). Overall, females have the highest risk of mortality if they do not lay eggs and have been in amplexus for at least 3.5 weeks, regardless of hormone treatment. Interestingly, females that did not lay eggs *and* were not hormonally treated after 2 weeks of amplexus, had a significantly higher mortality rate (χ^2^ (3, 209) = 26.949, *p* < .00001) than females that were treated with GnRHa, and did not lay eggs. Furthermore, within the GnRHa treated group, mortality was similar whether females oviposited (16.3%; *n =* 8/49) or not (29.3%; *n =* 12/41), (χ^2^ (1, 90) = 2.163, *p* = .14). Moreover, the mortality rate was similar for frogs treated with any of our GnRHa concentrations tested, regardless of number of pulses (χ^2^ (3, 90) = 0.982, *p* = .80). Post- spawning deaths ranged from 9.1–25.0% regardless of GnRHa concentration. Females that did not oviposit at all had the highest proportion of fatalities across all GnRHa treatment groups, 37.5, 23.1, 22.2 and 36.4% for 0.5, 0.1, 0.2 and 0.4 μg/g BW respectively, but there was no significant effect of concentration (χ^2^ (3, 41) = 1.028, *p* = .79). As a result, the chances of a female frog dying is independent of whether the female was hormonally treated, the concentration administered, or the number of pulses given, while the reward of increased spawning using hormone therapy is significant.

**Table 2:** Mortality of female Panamanian golden frogs (*Atelopus zeteki*) after 2-weeks of natural breeding and in response to GnRHa treatments of 0.05, 0.1, 0.2, or 0.4 μg/g body weight. Data reflects the number and percent (%) of females that died after oviposition, and those that died without ovipositing

**Treatment**	**N**	**# Deaths (%) of Total N**	**Rate of Mortality (%)**	
			Laid then died	Death if no eggs laid
No-hormone	111	20/111 (18.0)	12/102 (11.7)	8/9 (89.0)
All GnRHa	90	20/90 (22.2)	8/49 (16.3)	12/41 (29.3)
*P*-value	–	0.46	0.44	0.00008
**GnRHa μg/g BW**	**N**	**# Deaths (%)**	Laid then died	Death if no eggs laid
0.05	23	5/23 (21.7)	2/15 (9.1)	3/8 (37.5)
0.10	25	5/25 (20.0)	2/12 (10.0)	3/13 (23.1)
0.20	22	4/22 (18.2)	2/13 (18.2)	2/9 (22.2)
0.40	20	6/20 (30.0)	2/8 (25.0)	4/11 (36.4)
*P*-value	–	0.80	0.91	0.87

### Experiment 2: effect of dopamine antagonist

We tested if there was an effect of adding 150 μg of MET to 4 μg GnRHa on either oviposition or mortality rates of female PGFs compared to GnRHa alone. All females in this experiment were a grade 5 on the gravidity scale. Overall, we found that of the 84 female frogs treated with either GnRHa (*n =* 50) or GnRHa + MET (*n =* 34) there was a higher proportion of spawning than the background control rate of natural breeders (χ^2^ (1,515) = 15.460, *p* = .00008). Interestingly, there was no additional effect of adding MET to the GnRHa, (χ^2^ (1,84) = 0.029, *p* = .86) compared to spawning numbers for GnRHa alone (Table 3). A single pulse of GnRHa alone successfully induced 36.0% (*n =* 18/50) of all treated females to release eggs, compared to 26.4% from GnRHa + MET (*n =* 9/34). The second pulse of GnRHa caused 10.0% (*n =* 5/50) more females to spawn while a second pulse of GnRHa + MET induced an additional 17.7% (*n =* 6/34) to spawn. Importantly, over half of all females failed to spawn, even after two pulses of exogenous hormones at these concentrations (mean GnRHa = 0.25 μg/g BW), which is in line with the spawning rate observed in Experiment 1 for a GnRHa dosage between 0.2–0.4 μg/g BW. Overall, adding the dopamine antagonist MET did not result in any more female PGFs to spawn beyond the response rate of GnRHa alone.

**Table 3:** Oviposition by female Panamanian golden frogs (*Atelopus zeteki*) given GnRHa or GnRHa + metoclopramide treatments. Data reflects the number of females that laid eggs and percent (%) at each stage of the process

**Treatment**	**N**	# **Females Ovipositing (%)**			**Did not lay**
No hormone	431	102 (23.7)			329 (76.3)
Hormone	84	38 (45.2)			46 (54.8)
P-value	–	0.00008			–
**Hormone**	**N**	**# Females Ovipositing (%)**	**Pulse Response (%)**		**Did not lay**
			Laid w/ 1 pulse	Laid w/ 2 pulses	
GnRHa	50	23 (46.0)	18 (36.0)	5 (10.0)	27 (54.0)
GnRHa + MET	34	15 (44.1)	9 (26.4)	6 (17.7)	19 (55.9)
P-value	–	0.86	0.36	0.42	0.86

**Fig. 2:**
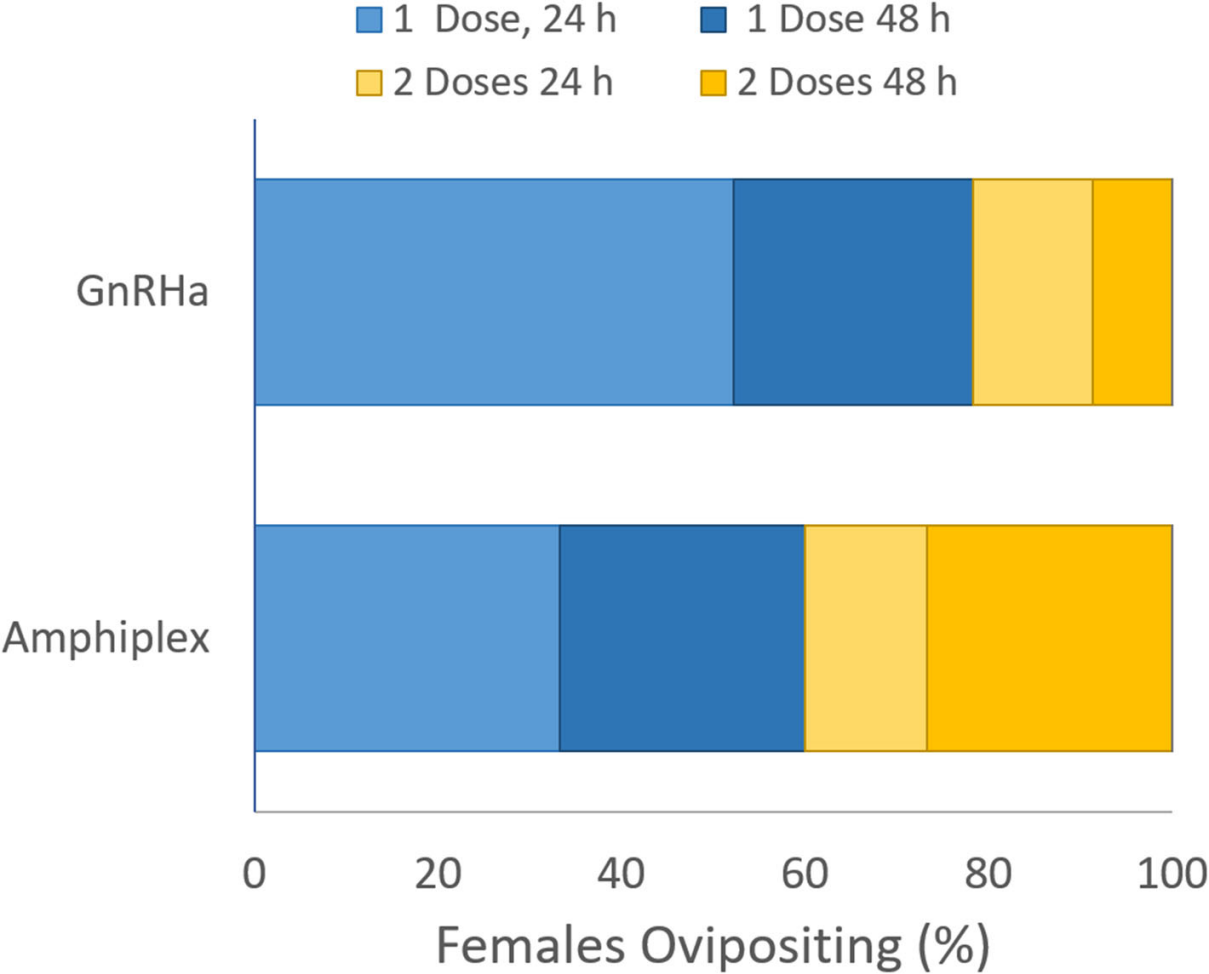
Within the group of ovipositing female PGFs, the latency in days to oviposition after one (blue shades) or two (gold shades) pulses of GnRHa alone or GnRHa + metoclopramide

Within the sub-population of spawning females there was an interesting distribution of the latency to oviposition between GnRHa alone and GnRHa + MET (Fig. 2). Females that spawned in response to GnRHa alone (*n =* 23) did so at a constant rate over time, with half of all remaining females that were going to spawn doing so at each 24 h period. Specifically, 52% of all spawning females oviposited < 24 h, the next 26% from 24 to 48 h, the next 13% between 48 and 72 h and the final 9% of spawning females laid between 72 and 96 h. No females responded after 96 h from the initiation of hormone therapy. Overall, 78% of all GnRHa responsive females laid eggs after the first pulse, and the second pulse induced the final 22% of laying females to oviposit. By contrast, the latency to ovipositon with GnRHa + MET was more evenly distributed over time such that the proportion of females laying in each 24 h period was 33, 27, 13 and 27%, respectively. Only 60% of all laying females responded to the first pulse of the GnRHa + MET compared to 78% with GnRHa alone, which was significantly lower (χ^2^ (1, 200) = 7.574, *p* = .0059) when considered on a proportional basis (Fig. 2). However, this also means a larger fraction (40%) of spawning females needed the second pulse of GnRHa + MET to complete the spawning process. Additionally, more females laid eggs in the 72–96 h window when treated with GnRHa + MET (27%) compared to GnRHa alone (9%). Thus, although PGFs eventually respond similarly to GnRHa whether or not supplemented with MET, the latency period to obtaining eggs from spawning females is shorter with GnRHa alone.

Compared to the background mortality rate (18.0%), there was no effect to adding the MET (χ^2^ (2,195) = 0.779, *p* = .67), (Table 4). Moreover, the mortality rate was independent of the number of pulses of hormones or latency period when frogs laid eggs naturally, with GnRHa alone or with MET supplementation (χ^2^ (2,140) = 4.420, *p* = .109). Thus, there is no apparent detrimental effect to female frogs’ health with respect to the addition of MET to the GnRHa treatment, nor with respect to the number of pulses that included additional MET. As seen previously (Table 2), there was a significant increase in the number of female PGFs that survived even if they did not oviposit, (χ^2^ (2,63) = 17.155, *p* < .0002) if the females were give GnRHa alone or with MET, compared to those left untreated.

**Table 4:** Mortality of female Panamanian golden frogs (*Atelopus zeteki*) given GnRHa or GnRHa + MET treatments. Data reflects the N of treated females and percent (%) that laid eggs and then died, as well as those that died but did not lay eggs

Treatment	N	# Deaths (%) of Total N	Rate of Mortality (%)	
			Laid then died	Death if no eggs laid (%)
No hormone	111	20/111 (18.0)	12/102 (11.7)	8/9 (89.0)
GnRHa	50	12/50 (24.0)	6/23 (26.1)	6/27 (22.2)
GnRHa + MET	34	7/34 (20.6)	4/15 (26.7)	3/19 (15.8)
P-value	–	0.67	0.11	< 0.0002

## Discussion

With the presumptive disappearance of the wild population of Panamanian golden frogs in the past decade, having a sustainable captive population is critical to the long-term survival of the species, especially with the goal to eventually release animals back into the wild with maintained genetic diversity. Thus, the decline in natural reproduction over the last decade and high mortality rate during the breeding season has necessitated the use of exogenous hormones to stimulate spawning in PGFs. Results from this study reveal that GnRHa significantly increased the number of spawning females compared to those that were allowed to breed naturally prior to hormone administration. Moreover, of the GnRHa concentrations tested, all induced spawning with similar response rates, and combining GnRHa with MET did not increase the number of spawning females compared to GnRHa alone. Fortunately, the hormone treatments did not present higher levels of mortality compared to background rates that occur during natural spawning. Although we did not find that hormone treatments were a panacea for inducing all females to spawn or for reducing mortality loss when females laid eggs, it does appear to make a significant difference in non-spawning female survivability. Additional research is needed to increase overall spawning rates, but hormone treatments may significantly decrease mortality during the breeding season of egg-bound females. Altogether, the hypotheses that increasing GnRHa concentrations, supplementing with MET, or administering additional pulses of hormones would induce substantially more females to ovulate over time, were rejected. These findings provide new knowledge on female PGF reproductive physiology and impact best management practices for the captive propagation and conservation of this critically endangered species.

Oviposition depends on the degree of oocyte development, which is a function of species-specific biotic and abiotic cues related to the environment, nutritional plane, hibernation stimuli or combinations of these factors. The stages of oogenesis [41], can be estimated by visual observation through non-invasive methods such as candling, gravidity scaling, and more recently ultrasound imaging [14, 15, 24, 42–44]. A level 4 and 5 on the gravidity scale for female PGFs generally parallels the secondary growth phase (stages 4–6) and final maturation (stage 7) of oogenesis, respectively [41].

Female PGFs are thought to be ready for breeding when they reach grade 5 based on anecdotal observations, reflecting a prominent egg mass of large oocytes that significantly displace the female’s organs by filling a majority of the abdomen, which becomes distended [15]. Similarly, in the dusky gopher frog, a grade 5 ultrasound image is associated with the female’s body cavity exhibiting a distended abdomen and sides due to enlarged ovaries and mature eggs [24]. Notably, pulsed hormone stimulus in ultrasound grade 3 and 4 female dusky gopher frogs promoted advancement of ovarian state to stage 5 and resulted in subsequent spawning [24]. For the PGFs, we found no difference in the spawning rates from females at gravidity scale 4 or 5, regardless of the hormone concentrations tested, number of hormone pulses given and no difference in the latency to spawning. Most PGF females were a grade 5 (77.8%) before being paired with a male, and thus should have had mature eggs ready to oviposit in the 2 week period of natural breeding followed by hormone therapy. Females of grade 4 (22.2%) may have had their egg masses mature to grade 5 in response to male amplexus prior to hormone treatment (data not collected). If males begin amplexing females prior to the full development of the egg mass, amplexus may be a requirement for completion of the oocyte developmental processes through secondary growth and final maturation before spawning, which could explain the unusually long amplexus times seen in the PGF compared to other species. In total, just over half of the female PGFs spawned despite the advanced degree of egg development, indicating components of the breeding process, whether biotic or abiotic, are still unaccounted for in the captive breeding environment.

This is the first study to determine if a dose response relationship was present between increasing concentrations of GnRH and spawning rates in PGFs, with the goal of developing an optimized breeding protocol. Findings from this study suggest a dose of 0.05 μg/g BW GnRHa induces oviposition at comparable rates as higher doses in female PGFs. Similar low doses of GnRHa (0.1 μg/g) have successfully induced ovulation in several Bufonidae species including the Puerto Rican crested toad [37] and boreal toad (*Anaxyrus boreas boreas*) [27]. However, other more distantly related anuran species require doses as high as 5 μg/g BW to induce [20, 22, 36]. This amount of GnRHa is more than 12 times the concentration on a per gram BW basis than the maximum of 0.4 μg/g BW typically given in bufonid species, and more than 100 times greater than the minimally effective dose of 0.05 μg/g BW determined here for inducing oviposition in the Panamanian golden frogs. Not only were female PGFs responsive to very low GnRHa concentrations, there was no statistically significant trend observed with increasing concentration of GnRHa administered in a single dose across the range 0.05–0.4 μg/g BW. One interpretation for lack of a dose response curve is that the lowest concentration of GnRHa saturated the majority of pituitary receptors, limiting the response of higher GnRHa concentrations stimulating the HPG-axis.

The mechanistic response to GnRHa and initiation of the hormone cascade depends on the number, and binding efficiency, of hormone receptors and their patterns of expression, which may vary across amphibian species. Several investigators studying hormone response in amphibians [31, 34, 45] have suggested too little exogenous hormone may not effectively induce receptor binding to stimulate the required signaling cascade, while too much may saturate and induce a down regulation of the receptors followed by desensitization to the hormone, rather than the desired outcome of increasing the downstream effects of the induced stimulation. The rate of clearance of GnRHa is unknown, allowing the possibility of a compounding effect of sequential treatments, so we also examined the ovulation response to a cumulative amount of GnRHa (0.05–0.8 μg/g BW) resulting from pulses of hormone treatments administered 48 h apart. We did observe a trend of decreasing rate of oviposition with increasing total hormone administered over the extended time period up to 96 h, although the trend was not statistically significant.

Roth et al. (2010) found that age, body size, and condition were all important factors that influenced the success of GnRHa effectiveness in induced spawning by boreal toads [27]. To determine if such factors effected oviposition in the PGF, this experiment used frogs with an average age of 5.7 years (range = 2–13 years old, mea*n* = 5.7, median = 5.6) and we distributed older age females (*n =* 5 females ≥8 years) evenly across treatments with all individuals having similar body weights (16.3 ± 0.5 g). Unlike the boreal toad, the PGF response to exogenous hormones appears to be independent of female age, weight or previous spawning history, similar to reports of hormone response in the dusky gopher frog [24], the southern corroboree frog [20], and Wyoming toad [19]. However, more recent changes in husbandry, and most notably nutritional state of the females, appear to also play a vital role in overall reproductive success in the PGF assurance colonies (Bronson, pers. observation).

Sequential administration of exogenous hormones has typically been discussed in terms of priming for improved fecundity and spawning rates of female amphibians [24, 26, 46]. Priming refers to administration of low doses of hormone 24–96 h prior to a resolving ovulatory dose, which is meant to stimulate the final stages of oocyte growth (stages 4–6) and maturation (stage 7) generating a grade 5 female ready to ovulate. A variety of hormones, including GnRHa, human chorionic gonadotropin (hCG) and progesterone, have been evaluated for their effectiveness in promoting oocyte growth and maturation, and usually the concentration of the priming doses are ~ 20% of the resolving ovulatory dose [25]. Female PGFs in this study were already at the oogenesis stage required for ovulation and did not clearly require priming hormones. Rather, the PGFs were treated with two hormone pulses at the resolving ovulatory dose to stimulate ovulation and oviposition. We found that a single pulse of GnRHa, at any concentration tested, caused complete oviposition by ~ 44% of all treated female PGFs. While some began to spawn within the first day, the majority laid eggs between 24 and 48 h after the initial hormone pulse. The latency to oviposition in the PGFs is similar to that observed where 85% of the egg clutches in the corroboree frogs were laid between 24 and 48 h post treatment [20, 46]. No partial clutches of eggs were observed after the first pulse.

We observed that a second pulse of GnRHa induced another ~ 10% of the treated females to lay eggs up to 96 h later, resulting in an overall ovulation rate of ~ 54%. While not significant, more females who were administered a lower concentration of GnRHa responded to the second pulse compared to the highest concentration, 17% vs. 5%, respectively, possibly suggesting receptor down regulation and desensitization at the higher concentrations. Anecdotal evidence is widely available within zoo and aquarium captive breeding programs, where multiple pulsed injections are given to female anurans; if they do not spawn on the first resolving dose it is a low probability that they will spawn after the second pulse, and almost no animals spawn after 3 hormone pulses. Although several papers have been published on the benefits of priming hormones [24] for female anurans that have yet to reach grade 4 or 5, very little has been published on pulsing hormones at resolving ovulatory concentrations. Despite the low rates of spawning in this study after two injections, when breeding genetically valuable individuals in an assurance population, it may in some cases be worth the additional handling of the frog for a second injection despite the lower likelihood of success in order to obtain eggs and avoid losing an individual female.

The purpose of supplementing GnRHa with MET, is to reduce the dopamine inhibition of the GnRH-induced release of LH and FSH to the gonads, effectively increasing the efficacy of GnRH binding. Previous studies on Northern leopard frogs advocated that the addition of MET to GnRHa promoted ovulation in females; however, there was no direct comparison to the GnRHa alone to determine the effect of MET supplementation [21, 38]. A direct comparison of GnRHa alone to GnRHa + MET was conducted in the dusky gopher frog, with results suggesting that the addition of MET to GnRHa was beneficial as it induced eight females to spawn compared to only five for GnRHa alone [24]. However, addition of MET to GnRHa in this species was no more beneficial than adding human chorionic gonadotropin (hCG) to GnRHa; cocktails of hCG and GnRHa have been demonstrated to induce ovulation in various anuran species [19, 20, 23–25, 47]. In the present study on PGFs, GnRHa yielded similar spawning rates and latency to egg laying as GnRHa + MET, suggesting GnRHa alone is sufficient to induce egg release by female PGFs. The absence of a downstream response from lifting the dopamine inhibition, suggests that the limited ovulatory response is due to more complex interactions regulating receptors and hormone cascades at the level of the gonads rather than at the pituitary.

Interestingly, male PGFs produced greater sperm concentrations when given GnRHa + MET compared to GnRHa alone [39]. However, there was not a direct comparison to this study on females, as the GnRH concentrations tested were much higher than those given to males in the GnRHa + MET combination. We found the lowest dose that induced spawning in our females was 100X times less than the highest GnRHa dosage Della Togna et al. (2017) tested for male PGF spermiation. Additionally, a nearly 10-fold increase in GnRHa did not stimulate more females to spawn, nor did the addition of MET, which acts to increase the effectiveness of GnRH by lifting the dopamine inhibition. It would be expected that intrinsic binding affinity by GnRH receptors, or other downstream receptors, could vary with evolutionary divergence, but that receptor expression or down regulation could vary between sexes of the same species. Differential gamete expression between the sexes, in response to treatment with GnRHa and/or dopamine antagonists, has been seen in certain lineages of temperate Australian frogs [48]. That review discussed the fact that although high rates of sperm release from Australian frogs were induced with GnRHa there was variability and inefficiency of the same GnRHa regimes for inducing gamete release from gravid females within the same taxa, especially for *Pelodryadid* tree frogs (spp. *Litoria*). Female PGFs ovulate and spawn moderately well (> 50%) when treated with GnRHa alone, similar to other species within the Bufonidae family including *Anaxyrus fowleri, A. baxteri, A. americanus, A. houstonensis, A. boreas boreas,* and *Peltophryne lemur* [19, 25, 27, 49] (*pers. comm.* Diane Barber) and thus reflect response patterns observed in the Australian ground frogs. Spawning and ovulation rates have been significantly advanced in recent years in several species of the family Bufonidae by selecting only females with advanced mature oocytes through ultrasound visualization [14, 24, 43, 44].

In addition to developing a standardized hormone protocol for spawning in PGFs, the following study was valuable in that it evaluated whether or not mortality during the breeding season could be reduced following treatment with GnRHa and oviposition. Hormone treatment did not appear to lower mortality rates beyond the established background rates for natural breeding when females successfully spawned; however, hormone treatment did decrease the mortality rate for females that did not oviposit after spending weeks in amplexus with a male compared to females that were not hormone treated. This study yielded a mortality rate between 18 and 30% regardless of GnRHa treatments or combination with MET, which was statistically different from background rates (89%) during natural breeding for non-ovipositing females. Of those females that were hormone treated, those that laid eggs had a mortality rate of 16% while 29% of those that didn’t lay eggs died after a month or more in amplexus, possibly due to egg-binding. These results suggest there was a benefit to stimulating females to lay eggs and that spawning provided some protection from egg binding and death. Sadly, very few studies using reproductive hormones report whether mortalities were observed, so we have little to compare our results within the published literature. Future research is needed to determine if additional hormones (e.g. oxytocin) or combinations of hormones can increase spawning rates above 60%, while decreasing mortality further.

## Conclusions and implications for management

PGFs exhibit a unique reproductive ecology with pairs remaining in amplexus for long periods of time [12], which in captivity has led to high rates of mortality not seen in other captive breeding programs. Here a gravidity scale has been described, whereby females exhibiting appropriate oocyte maturity can be selected for breeding and paired with a male. These efforts have significantly decreased mortality rates in the captive population, by choosing only females ready to ovulate. Moreover, we show that 1–2 pulses of GnRHa at 0.05 μg/g BW is sufficient to induce ovulation in > 50% of the females, the majority of eggs are ovulated following the first pulse, most of the females will ovulate in 48 h following hormone administration, and a subsequent pulse of hormone will stimulate a small percentage of additional females to lay eggs. Thus, caretakers can minimize the amount of hormone administered and separate amplexed animals after 48 h for better health management. Furthermore, mortality following hormone administration was decreased in females that failed to spawn. Although hormone treatment was not a cure-all for inducing all females to spawn, or completely correcting egg binding or the detrimental health effects from extended time in amplexus, it was beneficial in promoting survival rate of gravid females compared to those that remained untreated. Moreover, sharing information with the amphibian community that the death rate is not a function of hormone administration is valuable for adoption of these techniques. Equally important in successful egg-laying of females are environmental cues, such as the provision of optimal egg-laying habitats, underwater laying sites, and waterfall and misting elements, as well as nutritional health in the colony. The knowledge generated from this reproductive study has helped develop best management practices for the application of hormone therapy and assisted reproductive technologies for the critically endangered Panamanian golden frog.

## Methods

### Animals

Between 2001 and 2003, a North American breeding population of PGFs was established with the importation of 19 males, 19 females, and 12 juveniles of unknown gender. The Maryland Zoo in Baltimore has been intensively involved in the reproduction of this species, having produced > 2000 offspring that have been distributed around the country. PGFs managed in captivity are split into two closely managed populations by locale and phenotype and are managed by a Species Survival Plan (SSP). According to early field studies, females lay on average ~ 370 eggs in strings on the underside of rocks or underwater cavities in swiftly flowing streams [10]. Breeding cues provided by natural streams are replicated in captivity with fast water flow or the addition of a water cascade, whereby pumps for filtration aid circulation and speed providing a ‘waterfall’ effect. Field studies indicate females will lay eggs from November to January [10], but the breeding period varies based on different phenotypes and locations, and there can be two breeding seasons in some of the locations (Lindquist, *pers. comm*.). In captivity, female PGFs begin visible oogenesis around August, males will begin amplexus around October and egg laying can extend from November through May [16].

**Fig. 3:**
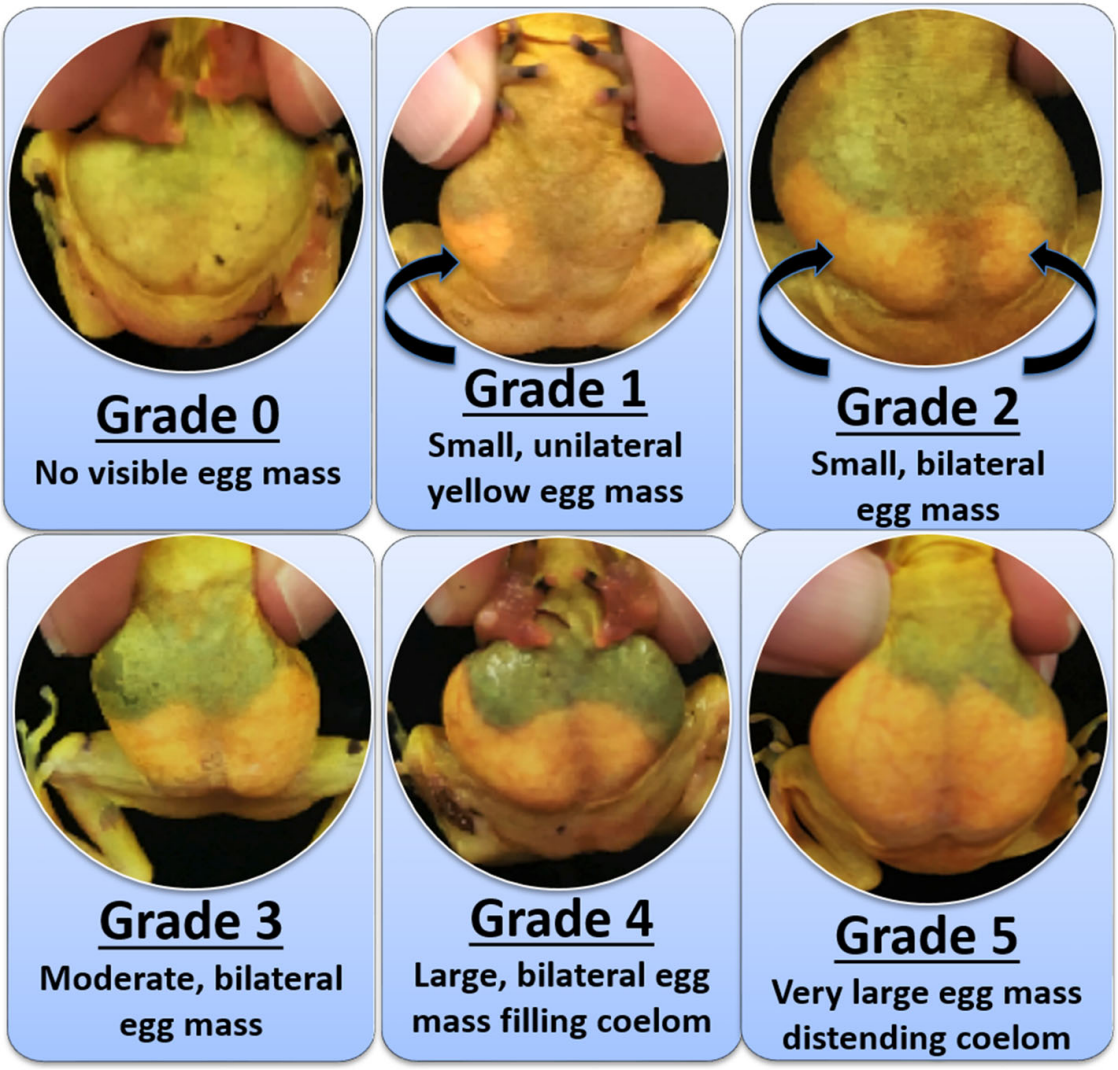
Gravidity scale of *Atelopus zeteki* indicating size of egg mass (light color) in coelom on a scale of 0–5 based on Bronson, 2015

Experiments were conducted at the MZIB during the 2014–15 and 2015–16 breeding seasons with adult female frogs (*n =* 174: age ≥ 2 years) from the F1-F3 generations of captive born PGFs. During the breeding season, females were regularly checked for egg development and added to the study groups as they reached a level 4–5 on the gravidity scale as previously described [14, 15], (Fig. 3). Upon reaching a gravidity level of 4–5, females were paired with a male and housed in a dedicated breeding tank (60 cm^3^) with a water depth of 30 cm and an elevated moss-covered platform (60 × 25 cm^2^). Terra cotta pots and faux plants provided cover on land and provided egg-laying sites under water. All paired frogs were allowed to breed naturally, and remained in amplexus until either the female oviposited or 2 weeks had passed. Females that do not naturally release eggs within 2 weeks are then administered exogenous hormones while the breeding pair remains in amplexus. There was no predetermined sample size for either experiment as the number of frogs undergoing hormone stimulation was unknown until female frogs developed adequate gravidity, and after the conclusion of the natural breeding attempt. All animals were returned to their standard enclosures after the end of the experiment. The MZIB’s Institutional Animal Care and Use Committee (IACUC) reviewed and approved all experimental procedures.

### Exogenous hormones

This project used two different hormone regimens, GnRHa alone or GnRHa + MET [21]. The GnRHa lyophilized powder ([Des-Gly 10, D-Ala 6]-LHRH ethylamide acetate hydrate; L4513; CAS Number 79561–22-1; Sigma-Aldrich, St. Louis, MO, USA) was reconstituted to a 20 μg/ml solution with distilled water and 0.9% saline in equal parts, aliquoted into cryovials and frozen at − 80 °C until shortly before use. Metoclopramide (Teva Parenteral Medicines, Inc., Irvine, CA, USA) was resuspended in a 0.7% saline solution as previously described and was added to GnRHa based on concentrations from Trudeau et al. (2010, 2013). All female frogs were injected intracoelomically with a 28 gauge needle and attached syringe (DG554801 1 cc U-100 Insulin Syringe, Becton Dickinson and Company, Franklin Lakes, NJ, USA), carefully avoiding the oocyte mass or other coelomic organs.

### Experiment 1: GnRHa dosage response

Experiment 1 was conducted to evaluate the optimal GnRHa concentration based on body weight (BW) needed to induce oviposition. Females (*n =* 90) that did not oviposit during the 2-week period of natural breeding were randomly assigned to one of four GnRHa treatment groups, 0.05 (*n =* 22), 0.1 (*n =* 25), 0.2 (*n =* 23), and 0.4 (*n =* 20) μg/g BW. The first hormone pulse was given in the morning and a second hormone pulse given 48 h later to any females that had not oviposited. Following each hormone pulse, breeding pairs were allowed to spontaneously spawn and tanks were checked daily for eggs. Breeding pairs that spawned were allowed to remain in amplexus until they naturally separated shortly after egg-laying was completed. Following the second hormone pulse, breeding pairs were allowed to remain in amplexus for up to 7 days. If spawning did not occur, they were manually separated, and individuals were closely monitored over the next 30 days for negative signs of health or mortality.

### Experiment 2: effect of dopamine antagonist

Experiment 2 compares the efficacy of GnRHa alone to GnRHa + MET for inducing oviposition in female PGFs. PGF females (avg. weight = 16.3 ± 0.5 g) were randomly assigned to a treatment group (*n =* 84/group) following 2 weeks of natural breeding attempts. Females were administered either 4 μg GnRHa (Treatment 1) or 4 μg GnRHa + 150 μg MET (Treatment 2) via intracoelomic injection while still in amplexus with the male as described above. For both treatments, the same concentration of hormone was applied to each female regardless of body weight in order to evaluate efficacy under standard breeding conditions; weight varied little for the females in our study. Using the average weight of females in the study, MET was administered at approximately 9.2 μg/g BW and GnRHa at 0.25 μg/g BW, similar to published recommendations [21]. A second pulse of the same hormone treatment was given 48 h later if the pair did not spawn. Amplexus was naturally discontinued if eggs were released in the tank, or animals were manually separated after a period of 7 days following the second hormone pulse. Subsequently, females were closely monitored over the next 30 days for any negative signs of health or mortality.

### Data analysis and statistics

Both experiments followed a completely random design with each female frog defined as an experimental unit, and the response variables oviposition and mortality treated as binary data, where a response was indicated as 1 and no response was indicated as 0. We measured the treatment response for hormone type (GnRHa or GnRHa + MET), hormone concentration (0–0.4 μg/g BW), the number of hormone pulses needed for a response (1 or 2), and latency period (delay from initial hormone administration to spawning). The latency period references the time since the initial hormone administration to oviposition and consisted of five categories: females that laid eggs in ≤24 h, 25–48 h, 49–72 h, 73–96 h, and 96 h to 7 days after initiation of hormone therapy. Females in the last three latency categories received a second pulse of the designated hormone treatment, thus latency in the present study is a categorical reference rather than a true time response. Only one female laid between 2 and 7 days after the second pulse in any experiment, so this category for latency was not included in the statistical analysis. Lastly, mortality refers to females that died within 30 days of the last hormone treatment received.

Response data was analyzed using a two-tailed (N-1) Chi-squared test (χ^2^) reported as: χ^2^ (degrees of freedom, *n =* sample size) = χ^2^ statistic value, *p = p* value, with results considered significant at an α <  0.05. Effects were evaluated against the control and between treatments. The control data was obtained from records collected between 2009 and 2016 on breeding females that were not treated with hormones, and females that spawned during the 2-week natural breeding period and/or died prior to hormone therapy. A Yates-correction was used in the analysis to account for the high number of females in the non-hormone treated reference set. In Experiment 1, which tested efficacy of GnRHa at 4 different concentrations, a 2 (observed binary responses) X 4 (treatments) contingency analysis was applied for each parameter: total females ovipositing, hormone pulse, and latency category, for each response variable (oviposition and mortality). Similarly, data in Experiment 2, which examined the effect of GnRHa + MET, was analyzed using a 2 X 2 contingency table for oviposition and a 3 × 2 contingency table for mortality. Statistical analysis was conducted using program R and JMP version 14.3.

## Data Availability

The datasets used and/or analyzed during the current study are available from the corresponding author on reasonable request.

## Abbreviations

Bd: *Batrachochytrium dendrobatidis*
BW: Body weight
GnRH: Gonadotropin releasing hormone
GnRHa: Gonadotropin releasing hormone analog
FSH: Follicle-stimulating hormone
h: Hour
hCG: Human chorionic gonadotropin
HPG axis: Hypothalamus-pituitary-gonad axis
IACUC: Institutional Animal Care and Use Committee
LH: Luteinizing hormone
MET: Metoclopramide
MZIB: Maryland Zoo in Baltimore
PGF: Panamanian golden frog

## References

[1] IUCN (2019). The IUCN red list of threatened species.

[2] Crawford AJ, Lips KR, Bermingham E (2010). Epidemic disease decimates amphibian abundance, species diversity, and evolutionary history in the highlands of Central Panama. Proc Natl Acad Sci U S A.

[3] Voyles J, Young S, Berger L, Campbell C, Voyles WF, Dinudom A (2009). Pathogenesis of chytridiomycosis, a cause of catastrophic amphibian declines. Science.

[4] Berger L, Speare R, Daszak P, Green DE, Cunningham AA, Goggin CL, Slocombe R, Ragan MA, Hyatt AD, McDonald KR, Hines HB, Lips KR, Marantelli G, Parkes H (1998). Chytridiomycosis causes amphibian mortality associated with population declines in rain forests of Australia and Central America. Proc Natl Acad Sci.

[5] Lips KR, Brem F, Brenes R, Reeve JD, Alford RA, Voyles J, Carey C, Livo L, Pessier AP, Collins JP (2006). Emerging infectious disease and the loss of biodiversity in a Neotropical amphibian community. Proc Natl Acad Sci U S A.

[6] Becker MH, Richards-Zawacki CL, Gratwicke B, Belden LK (2014). The effect of captivity on the cutaneous bacterial community of the critically endangered Panamanian golden frog (Atelopus zeteki). Biol Conserv.

[7] Gagliardo R, Crump P, Griffith E, Mendelson J, Ross H, Zippel K (2008). The principles of rapid response for amphibian conservation, using the programmes in Panama as an example. Int Zoo Yearb.

[8] Poole V (2008). Project golden frog. Endanger Species Updat.

[9] Poole V (2006). Panamanian golden frog husbandry manual.

[10] Karraker NE, Richards CL, Ross HL (2006). Reproductive ecology of Atelopus zeteki and comparisons to other members of the genus. Herpetol Rev.

[11] Lindquist ED, Sapoznick SA, Rodriguez EJG, Johantgen PB, Criswell JM (2007). Nocturnal position in the Panamanian Golden frog, Atelopus zeteki (Anura, Bufonidae), with notes on fluorescent pigment tracking. Phyllomedusa J Herpetol.

[12] Gawor A, Rauhaus A, Karbe D, Van Der Straeten K, Lötters S, Ziegler T (2012). Is there a chance for conservation breeding. Amphib Reptil Conserv.

[13] Lötters S (1996). The neotropical toad genus Atelopus: checklist, biology, distribution.

[14] Bronson E, Vance CK (2019). Anuran reproduction. Fowlers Zoo Wild Anim Med Curr Ther.

[15] Bronson E, Barrett K, Murphy K, Cranfield M (2015). Reproductive management of the Panamanian Golden Frog (*Atelopus zeteki*). 2015 Proceedings Annual Conference AAZV.

[16] Eustace R, Wack A, Mangus L, Bronson E (2018). Causes of mortality in captive Panamanian golden frogs ( Atelopus Zeteki ) at the Maryland Zoo in Baltimore, 2001–2013. J Zoo Wildl Med.

[17] Denardo D, Barten SL, Raiti P (2000). Dystocia.

[18] Feldman ML (2007). Some options to induce oviposition in turtles. Chelonian Conserv Biol.

[19] Browne RK, Seratt J, Vance C, Kouba A (2006). Hormonal priming, induction of ovulation and in-vitro fertilization of the endangered Wyoming toad (Bufo baxteri). Reprod Biol Endocrinol.

[20] Byrne PG, Silla AJ (2010). Hormonal induction of gamete release, and in-vitro fertilisation, in the critically endangered southern corroboree frog, *Pseudophryne corroboree*. Reprod Biol Endocrinol.

[21] Trudeau VL, Somoza GM, Natale GS, Pauli B, Wignall J, Jackman P (2010). Hormonal induction of spawning in 4 species of frogs by coinjection with a gonadotropin-releasing hormone agonist and a dopamine antagonist. Reprod Biol Endocrinol.

[22] Silla AJ (2011). Effect of priming injections of luteinizing hormone-releasing hormone on spermiation and ovulation in Gunther’s Toadlet, *Pseudophryne guentheri*. Reprod Biol Endocrinol.

[23] Clulow J, Clulow S, Guo J, French AJ, Mahony MJ, Archer M (2012). Optimisation of an oviposition protocol employing human chorionic and pregnant mare serum gonadotropins in the barred frog Mixophyes fasciolatus (Myobatrachidae). Reprod Biol Endocrinol.

[24] Graham KM, Langhorne CJ, Vance CK, Willard ST, Kouba AJ (2018). Ultrasound imaging improves hormone therapy strategies for induction of ovulation and in vitro fertilization in the endangered dusky gopher frog (Lithobates sevosa). Conserv Physiol.

[25] Guy EL, Martin MW, Kouba AJ, Cole JA, Kouba CK (2020). Evaluation of different temporal periods between hormone-induced ovulation attempts in the female Fowler’s toad Anaxyrus fowleri. Conserv Physiol.

[26] Browne RK, Li H, Seratt J, Kouba A. Progesterone improves the number and quality of hormone induced fowler toad (Bufo fowleri) oocytes. Reprod Biol Endocrinol. 2006;4(1):3. 10.1186/1477-7827-4-3. 10.1186/1477-7827-4-3 PMC1373633 16451718

[27] Roth TL, Szymanski DC, Keyster ED (2010). Effects of age, weight, hormones, and hibernation on breeding success in boreal toads (Bufo boreas boreas). Theriogenology..

[28] Vu M, Trudeau VL (2016). Neuroendocrine control of spawning in amphibians and its practical applications. Gen Comp Endocrinol.

[29] Tsai P-S, Norris DO, Lopez KH (2011). Neuroendocrine control of reproduction in amphibians. Hormones and reproduction of vertebrates. Volume 2: amphibians.

[30] Clulow J, Trudeau VL, Kouba AJ (2014). Amphibian declines in the twenty-first century: why we need assisted reproductive technologies. Adv Exp Med Biol.

[31] Silla AJ, Byrne PG (2019). The role of reproductive technologies in amphibian conservation breeding programs. Annu Rev Anim Biosci.

[32] Rastogi RK, Pinelli C, Polese G, D’Aniello B, Chieffi-Baccari G (2017). Hormones and reproductive cycles in anuran amphibians. Horm Reprod Vertebr.

[33] Kouba A, Vance CK (2009). Applied reproductive technilogies and genetic resource banking for amphibian conservation. Reprod Fertil Dev.

[34] Kouba AJ, Vance CK, Willis EL (2009). Artificial fertilization for amphibian conservation: current knowledge and future considerations. Theriogenology..

[35] Dorsey KM, Guthrie HD, Welch GR, Mohler J, Theisen DD, Siewerdt F, Vinyard BT, Woods LC (2011). Quality assessment of wild Atlantic sturgeon semen under conditions of short-term storage. N Am J Aquac.

[36] Michael SF, Buckley C, Toro E, Estrada AR, Vincent S (2004). Induced ovulation and egg deposition in the direct developing anuran Eleutherodactylus coqui. Reprod Biol Endocrinol.

[37] Crawshaw G. Veterinary participation in Puerto Rican crested toad program. In: Zoo and wild animal medicine: Elsevier; 2008. p. 126–36.

[38] Trudeau VL, Schueler FW, Navarro-Martin L, Hamilton CK, Bulaeva E, Bennett A, Fletcher W, Taylor L (2013). Efficient induction of spawning of northern leopard frogs (Lithobates pipiens) during and outside the natural breeding season. Reprod Biol Endocrinol.

[39] Della Togna G, Trudeau VLVLLVL, Gratwicke B, Evans M, Augustine L, Chia H (2017). Effects of hormonal stimulation on the concentration and quality of excreted spermatozoa in the critically endangered Panamanian golden frog (Atelopus zeteki). Theriogenology..

[40] Kouba A, Willis E, Vance C, Hasenstab S, Reichling S, Krebs J (2011). Development of assisted reproductive technologies for the endangered Mississippi gopher frog (Lithobates Sevosa) and sperm transfer for in vitro fertilization. Reprod Fertil Dev.

[41] Aranzabal MCU, Norris DO, Lopez KH (2011). Hormones and the female reproductive system of amphibians. Hormones and reproduction of vertebrates. Volume 2: amphibians.

[42] Johnson CJ, Vance CK, Kouba JA. Oviposition and ultrasound monitoring of American toads (*Bufo americanus*) treated with exogenous hormones: American Association of Zoo Veterinarians; 2002.

[43] Marcec R. Development of assisted reproductive technologies for endangered North American Salamanders: Mississippi State University; 2016.

[44] Reyer HU, Bättig I (2004). Identification of reproductive status in female frogs-a quantitative comparison of nine methods. Herpetologica..

[45] McDonough CE, Martin MW, Vance CK, Cole JA, Kouba AJ (2016). Frequency of exogenous hormone therapy impacts spermiation in male Fowler’s toad (Bufo fowleri). Reprod Fertil Dev.

[46] Silla A (2011). Effect of multiple priming injections of luteinizing hormone-releasing hormone on spermiation and ovulation in Guenther ‘s Toadlet, *Pseudophryne guentheri*. Reprod Biol Endocrinol.

[47] Wlizla M, Falco R, Peshkin L, Parlow AF, Horb ME (2017). Luteinizing hormone is an effective replacement for hCG to induce ovulation in Xenopus. Dev Biol.

[48] Clulow J, Pomering M, Herbert D, Upton R, Calatayud N, Clulow S, Mahony MJ, Trudeau VL (2017). Differential success in obtaining gametes between male and female Australian temperate frogs by hormonal induction: a review. Gen Comp Endocrinol.

[49] Kouba AJ, Lloyd RE, Houck ML, Silla AJ, Calatayud N, Trudeau VL, Clulow J, Molinia F, Langhorne C, Vance C, Arregui L, Germano J, Lermen D, Della Togna G (2013). Emerging trends for biobanking amphibian genetic resources: the hope, reality and challenges for the next decade. Biol Conserv.

